# Development of *Plasmodium falciparum* specific naïve, atypical, memory and plasma B cells during infancy and in adults in an endemic area

**DOI:** 10.1186/s12936-017-1697-z

**Published:** 2017-01-21

**Authors:** Allan Lugaajju, Sreenivasulu B. Reddy, Mats Wahlgren, Fred Kironde, Kristina E. M. Persson

**Affiliations:** 1 School of Biomedical Sciences, College of Health Sciences, Makerere University, Kampala, Uganda; 2 Department of Microbiology, Tumor, and Cell Biology, Karolinska Institutet, Stockholm, Sweden; 3 Habib Medical School, Islamic University in Uganda (IUIU), Kampala, Uganda; 4 Department of Laboratory Medicine, Lund University, Lund, Sweden

**Keywords:** *Plasmodium falciparum*, B-cells, Memory, Atypical, Immunity, Malaria

## Abstract

**Background:**

B-cells are essential in immunity against malaria, but which sub-sets of B-cells specifically recognize *Plasmodium falciparum* and when they appear is still largely unknown.

**Results:**

Using the flow cytometry technique for detection of *P. falciparum* specific (Pf+) B-cells, this study for the first time measured the development of Pf+ B cell (CD19+) phenotypes in Ugandan babies from birth up to nine months, and in their mothers. The babies showed increases in Pf+ IgG memory B-cells (MBCs), atypical MBCs, and plasma cells/blasts over time, but the proportion of these cells were still lower than in the mothers who displayed stable levels (5, 18, and 3%, respectively). Pf+ non-IgG+ MBCs and naïve B-cells binding to *P. falciparum* antigens were higher in the babies compared to the mothers (12 and 50%). In ELISA there was an increase in IgG and IgM antibodies over time in babies, and stable levels in mothers. At baby delivery, multigravidae mothers had a higher proportion of Pf+ IgG MBCs and less Pf+ naïve B-cells than primigravidae mothers.

**Conclusions:**

In newborns, naïve B-cells are a major player in recognizing *P. falciparum*. In adults, the high proportion of Pf+ atypical MBCs suggests a major role for these cells. Both in infants and adults, non-IgG+ MBCs were higher than IgG MBCs, indicating that these cells deserve more focus in future.

**Electronic supplementary material:**

The online version of this article (doi:10.1186/s12936-017-1697-z) contains supplementary material, which is available to authorized users.

## Background

*Plasmodium falciparum* malaria accounts for over half million deaths annually, with children being the most affected [1]. Children are the most vulnerable because malaria immunity is dependent on age and exposure [2, 3]. The blood stage of *P. falciparum* is responsible for most of the malaria-associated pathology. Disease symptoms range from fever to more severe complications, including respiratory distress, metabolic acidosis, renal failure, pulmonary edema and cerebral malaria. The clinical spectrum of symptomatic disease is caused by the asexual blood stages of *Plasmodium*, where the parasite undergoes cyclic replication within human erythrocytes [4]. Although the pathogenesis of malaria is not completely understood, it is believed to arise from the concerted effects of host and parasite factors, including the sequestration of infected erythrocytes in microvasculature, local and systemic inflammation [5]. Naturally acquired immunity is known to require antibody responses. The protective role of antibodies in combating malaria was first established by passive transfer of immunoglobulin G (IgG) from clinically immune adults into children with severe malaria, which rapidly attenuated the severity and burden of disease [6]. This has been supported by immuno-epidemiological studies, where antibodies to parasite antigens have been found to be associated with protection from clinical episodes in endemic areas [7–14]. Antibodies may limit the growth of blood-stage parasites and the development of clinical symptoms by several known mechanisms. These include blocking erythrocyte invasion [15–17], opsonising parasitized erythrocytes for phagocytic clearance [18, 19], monocyte-mediated antibody-dependent cellular killing [20, 21], and complement-mediated lysis [22], and in addition meddling with the adherence of infected erythrocytes to vascular endothelium [4]. Inadequate production of antibodies to *Plasmodium* antigens and their subsequent loss in the absence of persistent exposure has been proposed to impair B-cell immunological memory advancement [4]. Memory B-cells (MBCs) play an important role in durable resistance to different pathogens by boosting the immune response in times of secondary exposure. Studies have shown that antibody production can be sustained through re-stimulation of MBCs by persistent antigens [23] or by non-proliferating long lived plasma cells [24, 25]. Protection of the adult and the newborn is ensured by antibodies mostly of IgG and IgA isotypes. MBCs induced by natural infection or vaccination correspond to switched MBCs. In the peripheral blood, another population of MBCs, called IgM memory [26–28] has been described with different origin, function and significance. IgM MBCs, also known as natural memory or natural effector memory cells [29], develop in the absence of germinal centres [30], generate extra-follicular thymus-independent responses and produce natural antibodies [31]. Because of the host immature immune system and the antigenic variation of the malaria parasite, development of effective B-cells and antibody responses occurs after repeated years of exposure [32–36]. It has also been speculated that *Plasmodium* infection meddles with development and maintenance of B-cell memory response [37–41]. There is still need to fully understand the development, regulation and maintenance of immunity against malaria [36, 42, 43]. B-cell phenotypes created amid malaria bouts demonstrate the B-cells linked with malaria immunity development. Diverse research has portrayed numerous B-cell phenotypes in individuals exposed to different malaria episodes [35, 37, 38, 44–49]. Nahrendorf et al. [50] showed gradual acquisition of MBCs and antibodies recognizing pre-erythrocytic and cross-stage antigens after *P. falciparum* sporozoite immunization. However, the magnitude of these humoral responses did not correlate with protection but directly reflected parasite exposure in chemoprophylaxis and sporozoite immunization. In African youngsters after experiencing intense malaria, an expansion in both the total memory and transitional B-cell populaces was observed [51]. It is important to note that this earlier research studied the whole B-cell populace and did not estimate *Plasmodium falciparum* (Pf+) specific B cells. Elispot assay has been used to try and find parasite specific cells, for example to show that even if antigen-specific antibodies were not detected in plasma, antigen-specific B-cells could still be found circulating in the blood, suggesting that these could be maintained independently of long-lived plasma cells [52]. However, Elispot needs activation and survival of cells for a relatively long time, and compared to ELISA-based assays, flow cytometry is a good method for estimation of antigen-specific cells. While dealing with intricate antigens, flow cytometry has been shown to be a better assay option [53]. Malaria calls for flow cytometry analysis since it has a scope of parasite antigens that individually have a low number of specific B-cells. ELISA-based measures when improved can only quantify 70% of the response determined by flow cytometry [53]. Flow cytometry is advantageous in that there is no need of cell incitement thereby expanding the odds of incorporating all cells in the reading. In order to acknowledge how Pf+ B-cells are actuated and kept up in vivo, these cells should be isolated from other B-cells. Here, the flow cytometry technique for detection of Pf+ B-cells which was developed by Lugaajju et al. [54] was applied to monitor the development of Pf+ B-cell sub-populations in newborns from time of birth until 9 months and in their respective mothers, in a malaria endemic area.

## Methods

### Study site and subject enrolment

The study was conducted at Kasangati Health Centre (KHC), a referral unit of Wakiso district which is located 20 km north east of Kampala, the capital city of Uganda. The antenatal clinic of KHC is a public charge-free facility that runs 5 days a week. On average, 60 patients (20 new visits, 40 revisits) are seen per day and about 7 deliveries occur daily. In this study area, malaria is meso-endemic with peak transmission after the two rainy seasons (February–March and September–October) every year. The study region is peri-urban and over 90% of pregnant women attend the antenatal clinic at least once. Between March of 2012 to July of 2013, patients were recruited in their last trimester from peri-urban villages within 20 km from KHC. Eligibility criteria were: Normal deliveries with healthy newborns, agreement to come to the study clinic for follow up at 10 weeks, 6 and 9 months of the child’s age. The selection of the study participants was random (patients were selected sequentially as they came to the clinic unless they failed the inclusion criteria). As policy at KHC, every pregnant woman took at least one or two does of IPT during the pregnancy of the delivered baby. Also, every pregnant woman was given a long-lasting insecticide mosquito bed net. During recruitment and follow up visits, a detailed clinical examination was performed and the data were entered into the study questionnaire. Malaria rapid diagnostic test (RDT) and blood smear examination upon a positive RDT were performed.

### Sample collection and processing

On the day of baby delivery, 5 to 10 mL of mother’s venous blood and the respective umbilical cord blood of the newborn baby were collected and mixed with lithium heparin (BD, Plymouth, UK). At follow-up days, 2 to 4 mL blood from babies and 5 to 10 mL from respective mothers (at 9 months) were collected. Within 4 h after being collected, the blood samples were transported to the Makerere University, Biomedical Cross Cutting Laboratory for processing. Peripheral blood mononuclear cells (PBMCs) were separated by density gradient centrifugation using Ficoll-hypaque (GE HealthCare Bio-Sciences AB, Sweden). For this, the blood specimen was diluted with equal volume of Dulbecco’s phosphate buffered saline (DPBS, Life technologies, Stockholm, Sweden), carefully layered over the Ficoll, and centrifuged (400 g) for 30 min at room temperature. After centrifugation, plasma was removed and stored at −80 °C. The PBMCs were then collected, washed twice with DPBS (300 to 400 g for 15 min × 2) to remove Ficoll traces and platelets. The cells were then suspended in 1 mL RPMI medium (Sigma, St Louis, MO) stained with 0.4% trypan blue (w/v) and counted in a Neuberger chamber. The PBMCs were then cryopreserved in liquid nitrogen at concentration of 10^7^ cells/mL in heat-inactivated 90% fetal bovine serum (Sigma, St Louis, MO) and 10% DMSO (v/v) (Sigma, St Louis, MO) as previously described [55].

### Malaria diagnostics

All samples were tested by pLDH/HRP2 rapid diagnostic test (RDT) strips (Combo Rapid Diagnostic Test of Premier Medical Corporation Limited, India) as described by Bharti et al. [56]. Thick blood smears were stained with 10% Giemsa dye for 10 min, *Plasmodium* spp blood stage parasites were then counted in microscope fields containing at least 200 white blood cells (WBCs). The parasitaemia was calculated according to the WHO guidelines [57].

### Measurement of total anti-*P. falciparum* IgG and IgM by ELISA

Pf+ total IgG and IgM in blood plasma were measured by enzyme linked immune-sorbent assay (ELISA) as described [58]. Briefly, microtiter plate wells were coated with 1 µg of schizont extract per well (overnight at 4 °C), and blocked with 5% skimmed milk (Sigma) for IgG and super block dry blend (Thermo Scientific) for IgM for 2 h at room temperature. Plasma specimens were diluted 1:200 with plasma dilution buffer (2.5% milk powder in phosphate buffered saline with tween 20 plus 0.02% sodium azide). Diluted plasma samples were added to the wells in quadruplets and incubated in the microtiter plates at room temperature for 1 h. The microtiter wells were washed 4 times between the incubation (coating, blocking, first and secondary antibody) steps. The wells were then incubated (45 min) with diluted (1:20,000) peroxidase-conjugated goat anti-human IgG/IgM (Sigma) and rewashed. Bound secondary antibody was quantified by adding TMB (3, 3′, 5, 5′-Tetramethylbenzidine) substrate (Promega). Optical density (OD) was read at 450 nm with a reference at 620 nm. Plasma samples of Swedish individuals unexposed to *P. falciparum* infections were used as negative controls. All specimens were analysed twice and the means of the ELISA OD used in the analysis.

### *Plasmodium falciparum* ghost infected red blood cells (GiRBCs)—carboxyl Qdot conjugation

Red blood cells were infected with the FCR3S1.2 *P. falciparum* strain and maintained in vitro as described by Beeson et al. [59]. The cultures were synchronized using 5% D-sorbitol (Sigma) in water. The magneticallly enriched trophozoite pellet was treated with streptolysin O (Sigma) to obtain GiRBCs as described in Methods in Malaria Research [60]. The GiRBC (225 µg) was conjugated with 2 nmol carboxyl Qdot (35 µL) using freshly prepared 10 mg/mL *N*-ethyl-*N*-dimethylaminopropyl-carbodiimide as described by Lugaajju et al. [54]. The carboxyl Qdot-GiRBC conjugate was diluted 10 times with 10 mM Borate buffer (pH 7.4) and stored at 4 °C.

### Immunophenotyping of *P. falciparum* specific B-cells

Immuno-phenotyping of *P. falciparum* was done according to the protocol previously described by Lugaajju et al. [54]. Cryopreserved PBMCs (approximately 1 × 10^6^ cells) were thawed on ice and washed in cold flow buffer (PBS/0.5% BSA/2 mM EDTA). Flow buffer (100 μL) was added to each PBMC sample followed by 1 µg Fc block (CD16/CD32 mAb, Biolegend) and incubated at 0 to 4 °C (on ice water) for 5 min to minimize non-specific binding and background fluorescence. Carboxyl Qdot-GiRBC Conjugate (25 µL) was added, incubated on ice for 30 min and the cells were re-washed. Eight microliters of fluorochrome (FITC)-conjugated mouse anti-human IgG monoclonal antibody (mAb, BD Horizon) were added and incubated for 30 min to stain 10^6^ cells in 100 µL of flow buffer. The cells were then washed and stained for 30 min with 3.5 µL each of CD 19 PE CF594, CD20 V450, and FcRL4 APC fluorochrome-conjugated mouse anti-human mAb (BD Horizon). After the staining, the cells were washed and re-suspended in 300 µL flow buffer. The analysis was done on a LSRII flow cytometer (Becton–Dickinson Immuno Cytometry Systems, San Jose, USA). Data was processed using FLOWJO software (Tree Star Inc., San Carlos, and Ca, USA).

### Statistical analysis

Data analysis was carried out using STATA and Graph pad prism. Changes in IgG, IgM levels and Pf+ B-cell phenotypes for babies and mothers over time were assessed with Wilcoxon rank sum test. The differences in medians between two time points were evaluated by Mann–Whitney test and non-parametric Kruskal–Wallis test was used for comparing more than three time point.

## Results

### General characteristics of the study population

Healthy newborns (n = 131) and their respective mothers were enrolled into the study. However, during the nine months of post-natal follow-up, only 109 mother–baby pairs fulfilled all the follow-up time points (birth, 10 weeks, 6 and 9 months) and were included in the subsequent analysis. The mean age of the enrolled mothers was 25 years and ranged from 18 to 39 years. Of the 109 mothers, 27% were primigravidae and 73% mutigravidae. The parasitaemia as determined from the samples that were RDT positive at the different follow-up time points is shown in Table 1.

**Table 1:** Showing parasitaemia (number of infected RBC/µL of blood) for mothers and babies at different time points

Time course	Pat ID	Parasitaemia
Mother at birth		
	KM 12	48
	KM 32	16
	KM 35	240
	KM 41	64
	KM 58	32
	KM 60	2760
Baby at birth		
	KB 101	13,000
Baby at 2.5 months		
	KB 120 A	9560
Baby at 6 months		
	KB 56 B	91,760
	KB 118 B	22,6240
	KB 125B	3840
Baby at 9 months		
	KB 57 C	5080
Mother at 9 months		
	KM 91 C	1520
	KM 96 C	800

### Development of IgG and IgM antibody responses to *P. falciparum*

**Fig. 1:**
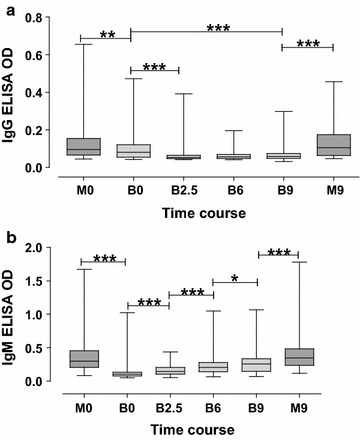
Development of IgG and IgM antibody responses to *Plasmodium falciparum.* The development of IgG and IgM antibodies in plasma against *P. falciparum* schizont extract was analyzed by ELISA using plasma specimens of mother–baby pairs (n = 109). The graphs show levels of IgG (graph **a**) and IgM (graph **b**) in plasma of blood collected from babies at birth, 2.5, 6 and 9 months later (see coordinate axis labels B0, B2.5, B6 and B9) and from mothers at delivery and 9 months later (M0 and M9). There was no difference in the levels of IgG and IgM for mothers at birth and 9 months later. At birth, babies had lower levels of IgG (**a**) and IgM (**b**) as compared to their mothers. Levels of IgG and IgM in babies increased over time although they did not reach the levels prevailing in the mothers’ blood at 9 months after baby delivery. The* horizontal lines* in the* box plots* with* whiskers* from minimum to maximum show median levels. *Asterisk*, *double and triple asterisks* indicate significant differences (p < 0.05, 0.01 and 0.001, respectively) between groups as evaluated by Wilcoxon rank sum test

To determine the development of plasma IgG and IgM antibodies against *P. falciparum,* schizont extract was used in ELISA. The Swedish samples were used as negative controls and their average antibody OD values for IgG (OD = 0.046) and IgM (OD = 0.09), were subtracted from the corresponding raw data of Ugandan specimens. There was no difference between levels of IgG for mothers at delivery and 9 months later. Babies at birth had slightly lower levels of Pf+ IgG compared to their mothers. These IgG levels in babies decreased even further at 2.5 months, and then increased by 6 and 9 months, but were still lower than the corresponding levels in their mothers (Fig. 1a). Similarly, there was no difference in levels of Pf+ IgM for mothers at delivery compared to 9 months later. At birth, babies had very low levels of anti-Pf IgM but these levels increased throughout the time points up to 9 months even though they did not reach the levels present in their mothers (Fig. 1b). In the mothers, there was no correlation between age and antibody titer levels.

### Development of *P. falciparum* specific B-cell sub-populations

**Fig. 2:**
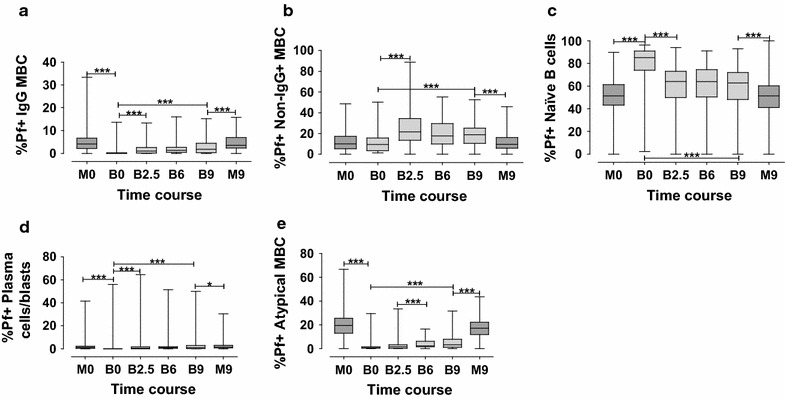
Development of *Plasmodium falciparum* specific B-cell sub-populations. Proportions of Pf+ CD19^+^B-cell sub-sets in mothers and babies: IgG MBCs (**a**), non-IgG+ MBCs (**b**), naïve B-cells (**c**), plasma cells/blasts (**d**) and atypical MBCs (**e**) determined using quantum dots flow cytometry. The coordinate axes show time points (as in Fig. 1) at which the tested blood specimens were collected

In order to assess the development of different fractions of Pf+ B-cells, five B-cell sub-sets were characterized from whole peripheral blood collected at the time of delivery (M0 = mother, B0 = baby at birth), baby at 2.5 months (B2.5), baby at 6 months (B6), baby and mother at 9 months (B9, M9 respectively). The flow cytometry technique for detection of B lymphocytes (defined as CD19+ cells) that are Pf+ was used to measure the relative proportions of IgG MBCs (CD19+CD20+CD27+FcRL4±IgG+), non-IgG+ MBCs (CD19+CD20+CD27+FcRL4±IgG−), naïve B-cells (CD19+CD20+CD27−FcRL4±IgG−), plasma cells/blasts (CD19+CD20−CD27+FcRL4±IgG−), and atypical MBCs (CD19+CD20+CD27−FcRL4±IgG+) as shown in Fig. 2. In the mothers, the mean proportions of Pf+ IgG MBCs, non-IgG+ MBCs, naïve B-cells, plasma cells/blasts and atypical MBCs were 5, 12, 50, 3 and 18% respectively. For mothers, there was no significant difference between the proportions of any of the Pf+ B-cell sub-populations at delivery compared to 9 months later. For the babies, there was an increase over time in proportions of Pf+ IgG MBCs, non-IgG+ MBCs, plasma cells/blasts and atypical MBCs. Although the proportions of these cells increased, the percentages did not reach adult levels for any of them, except for the non-IgG+ MBCs where levels were actually higher in the babies compared to the mothers. At birth, the B-cells recognizing *P. falciparum* in the babies were dominated by the naïve B-cells. Even though the proportions of Pf+ naïve B-cells decreased over time in babies, at 9 months of age the ratios of these Pf+ naïve B-cells were still higher in the infants compared to the mothers.

### Levels of anti-schizont IgG and IgM in high and low *P. falciparum* specific B-cell responders

**Fig. 3:**
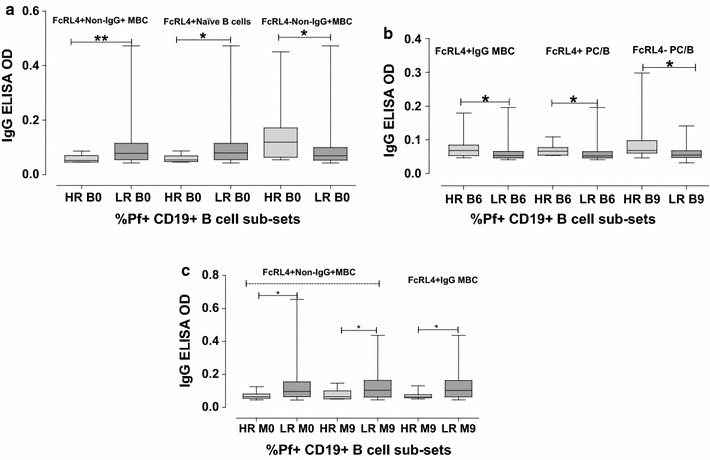
Levels of antibodies in IgG ELISA. Measured against schizont extract for individuals that were high responders (HR: top 10% of values) compared to low responders (LR: lowest 90% of values). **a** Babies at birth, **b** Babies at 6 and 9 months, **c** mothers at birth and 9 months later. Markings as in Fig. 1

**Fig. 4:**
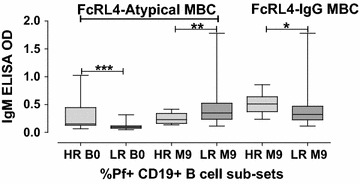
Levels of antibodies in IgM ELISA. Measured against schizont extract for individuals that were high responders (HR: top 10% of values) compared to low responders (LR: lowest 90% of values). Markings as in Fig. 1

The flow cytometry percentage frequency data for each Pf+ CD19+ B-cell sub-population was arranged in descending order. The top 10% of the values were considered the high responders and the lowest 90% of the values were considered as the low responders. Comparisons of the high responders to low responders (lowest 90% of values) for the sub-populations of Pf+ CD19+ B-cells were made and correlated to the levels of plasma anti—*P. falciparum* IgG and IgM as determined by ELISAs. Those that showed significant differences between groups of individuals are shown in Figs. 3 and 4 for IgG and IgM, respectively. For babies at birth, low proportions of Pf+ B-cell sub-populations including FcRL4+non-IgG+MBCs (CD19+CD20+CD27+FcRL4+IgG−) and FcRL4+ naïve B-cells (CD19+CD20+CD27−FcRL4+IgG−) were associated with high levels of schizont specific plasma IgG, well as high levels of FcRL4−non-IgG+MBCs (CD19+CD20+CD27+FcRL4−IgG−) were associated with high levels of schizont specific plasma IgG (Fig. 3a). High levels of FcRL4+IgG MBCs (CD19+CD20+CD27+FcRL4+IgG+), FcRL4+ plasma cells/blasts (CD19+CD20−CD27+FcRL4+IgG−) and FcRL4− plasma cells/blasts (CD19+CD20−CD27+FcRL4−IgG−) were all associated with high levels of Pf+ plasma IgG for babies at 6 and 9 months respectively (Fig. 3b).

For the mothers, low proportions of Pf+ B-cell sub-populations including FcRL4+ non-IgG+MBCs (CD19+CD20+CD27+FcRL4+IgG−) (at birth and 9 months), and FcRL4+IgG MBC (CD19+CD20+CD27+FcRL4+IgG+) at 9 months were associated with high levels of Pf+ plasma IgG (Fig. 3c). In addition, high proportions of FCRL4− Pf+ atypical MBCs (CD19+CD20+CD27−FcRL4−IgG+), and FCRL4− Pf+ IgGMBCs (CD19+CD20+CD27+FcRL4−IgG+) were associated with high levels of schizont binding plasma IgM for babies at birth and mothers at 9 months, respectively. However, low levels of FCRL4− Pf+ atypical MBCs were associated with high levels of IgM antibodies in mothers at 9 months (Fig. 4). Samples from malaria non-endemic areas (Swedish donors) showed less than 1% of each population of cells in flow cytometry, and also had very few numbers of cells in each subpopulation and hence no calculations could be made for valuable conclusions.

### Comparison of mother–baby parasitaemia with antibody and B-cell subpopulations

The respective parasitaemia for both mothers and babies at different follow up points were correlated with IgG, IgM Elisa ODs and the percentage frequency for each Pf+ specific CD19+ B-cell subpopulations. The results showed that increase in parasitaemia was associated with low IgG antibody OD values (*p* = 0.009). There was no significant correlation either between parasitaemia and IgM levels or between parasitaemia and the proportions different Pf+ specific CD19+ B-cell subpopulations. This could largely be due to the small numbers of Pf+ samples (10.7%).

### Comparison of primigravidae and mutigravidae

**Fig. 5:**
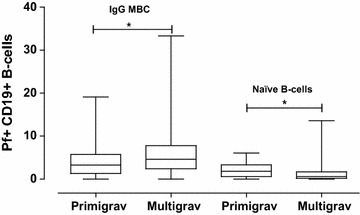
Comparison of primigravidae and mutigravidae. Comparison of proportions of Pf+ CD19+ IgG MBC and Pf+ naïve B-cells between primigravidae and multigravidae at birth. The *horizontal lines* show median levels in the* box plots*, with * whiskers* from minimum to maximum. *Asterisks* indicates significant differences (p < 0.05)

The study population comprised of primigravidae (27%) and mutigravidae (73%) mothers. Prominent differences were noted in proportions of Pf+ IgG MBCs and naïve B-cells at birth among primigravidae and mutigravidae as shown in Fig. 5. Pf+ IgG MBCs were higher in multigravidae than in primigravidae mothers (p < 0.05), whereas Pf+ naïve B-cells were higher in primigravidae compared to in multigravidae (p < 0.05).

## Discussion

In this study, a cohort of mothers and their newborn babies were recruited and followed up for 9 months after baby delivery. The development of plasma antibodies in the babies against schizont extract antigens showed a pattern that was what could be expected from individuals living in an endemic area. Schizont extract was used since it contains antigens from merozoites as well as other *Plasmodium* blood stages. The mothers showed stable levels of schizont specific IgG and IgM antibodies. The newborn babies had parasite specific IgG probably transferred through the placenta, followed by a decrease and then an increase of the IgG over time as the babies were exposed to malaria. This was also accompanied by an increase in IgM levels during the follow-up time for the babies. Similar results have been found before [61] and indicate that the people living in the study area are regularly exposed to malaria but also that full immunity is not achieved by 9 months of age. The initial antibody response to intrauterine infections in the newborns as determined in cord sera is generally of the IgM class [62]. In the present study, some infants had high levels of parasite specific IgM in the cord blood which suggests that they had been exposed to malaria parasites in utero, as was further indicated by one individual who actually had parasites in the cord blood. This baby also had a relatively high OD-value in IgM ELISA (0.3) compared to others at the same time point. The fairly rapid rise in the level of IgM as observed in the first 9 months of life reflects the primary immune response of the infants towards the malaria parasites, among other commonly known infections.

In addition, the study investigated the proportions of CD19+ Pf+ B-cells. In the mothers, the levels of these cells were stable, and showed IgG MBCs, non-IgG+ MBCs, naïve B-cells, plasma cells/blasts and atypical MBCs to be 5, 12, 50, 3 and 18%, respectively. In the literature, it has been assumed that IgG MBCs are the cells that are of importance for long-term memory in the defence against most diseases, but in this present study surprisingly high levels of non-IgG+ MBCs positive for Pf, both in the babies and in the mothers were found. These cells were negative for IgG, but most likely they could have been positive for IgM since memory cells recognizing IgD or IgE at these high levels would be unlikely because the total levels of these antibodies are normally very low. Moreover, IgA levels in blood directed against Pf have previously been shown to be very low [63] and sometimes even difficult to detect both in maternal and cord blood [64]. Acquisition of specific antibody isotypes to Pf antigens has in another study been shown to begin with IgM, followed by IgG1 and IgA [65] and the levels of the latter was shown to be detectable only in low levels and to increase not until after 9 months of age. IgA directed against Pf has been found in breast milk [66] but the levels are usually very low in blood [63]. IgM levels, on the other hand, have often been shown to be relatively high [67]. It is interesting to note that a relatively large proportion of the MBC pool is made up of non-IgG MBC. This is important information when trying to understand malaria immunity, and which parameters to focus on in vaccine studies.

It was also noted in this study that naïve B-cells binding to Pf were found to be at peak in the cord blood, which is in line with earlier studies showing naïve cells to be the major part of CD19+ response in newborns [68]. But even in our adult samples, about half of the cells showed binding to Pf which could indicate that in a normal immune response, these cells are relatively important. This signifies that besides IgG+ MBCs, other memory B cells (Pf+ non-IgG+ MBCs) are important in the defense against malaria.

In the present study, even though the proportions of the B-cell sub-sets increased in babies during the 9 months after birth, they were still lower than in their mothers, indicating that immunity is not yet achieved. In the mothers, there was no notable difference in the proportions of Pf+ IgG MBCs, non-IgG+ MBCs, atypical MBCs, plasma cells/blasts, and naïve B-cells at time of delivery and 9 months later, suggesting that the proportions of these circulating Pf+ B-cell sub-sets remains stable during adulthood. This finding is consistent with earlier studies of the whole B-cell pool [69], where the number of circulating B-cells remains stable during adulthood and it only decreases in individuals older than 60 years. Although based on murine models, it has been suggested that this is due to a reduced bone marrow ability to produce B-cells [70, 71]. In peripheral blood of healthy adult donors, human B-cells comprise very low numbers of plasma cells (1–3%) [69, 72] which is in line with the proportions of Pf+ cells found in the present study.

Among the B-cell sub-sets considered in this study, the atypical MBCs constituted a relatively large proportion. Individuals living in malaria-endemic areas were found with increased levels of atypical MBCs as age increased with cumulative *Plasmodium* exposure [35, 37, 38]. In this study, atypical MBCs were classified as CD19+CD20+CD27−IgG+, and in earlier studies, they have been characterized as CD10-CD19+CD20+CD21−CD27− [45, 73], suggesting that it is the same kind of cells. The proportions of atypical MBC has been shown to correlate with age and malaria transmission intensity [44, 74] and in the present study, the cells increase with age in the babies, but do not reach full adult levels during the 9 months of follow-up. Whether these atypical cells are good or bad for the immune defense is not completely clear, but considering the large proportion of Pf+ CD19+ cells that they constitute, they must be a major player in the development of immunity against malaria and they have previously been shown to express an array of inhibitory receptors as well as an impaired B-cell response [45].

In this study, cells and antibodies circulating in peripheral blood were investigated. Ideally, one would like to investigate bone marrow, spleen and lymph nodes as well to get a full picture of how immunity is formed, especially during the first year of life. However, for practical and ethical reasons this was not possible. Nevertheless, studies of peripheral blood cells and antibodies conceivably reflect what happens in the rest of the body and provides valuable insight.

Despite the fact that many individuals showed very low levels of some subpopulations of Pf+ cells, those that actually had higher levels of some specific cells compared to other individuals were interesting to investigate further. The study therefore compared the results for the high responders (top 10% of values) to the low responders (lowest 90% of values) for the subpopulations of Pf+ CD19+ B-cells, and also sub-divided the cells further into FCRL4+ and FCRL4− cells and correlated this to the levels of plasma IgG and IgM determined by ELISA. FcRL4 (CD307d) is exclusively present on B-cells and previous studies have proposed that FcRL4+ B-cells represent a specialized tissue sub-population of MBCs [75–78], hence they are capable of eliciting a secondary immune response. In order not to miss out on these special kind of memory B-cells, FcRL4 marker was included in the B-cell phenotyping panel to differentiate the MBCs. High levels of antibodies, especially IgG and IgM, have been shown in several previous studies to be associated with malaria immunity [11, 79]. In this study, the general pattern (Figs. 3 and 4) indicated that low proportions of FCRL4+ cells and high proportions of FCRL4− cells were associated with high levels of IgG or IgM antibodies. This could point towards a situation where it is better to not have too high a response of FCRL4+ cells. The FCRL4 marker has also been indicated to be of importance in HIV, which can induce an increase of FCRL4+ cells [80]. Interestingly, both HIV and malaria are infections that can persist for a long time in the human body (Additional file 1).

The study also compared primigravidae and mutigravidae individuals. It was noted that multigravidae had a higher proportion of Pf+ IgG MBCs and lower proportion of Pf+ naïve B-cells, as compared to primigravidae at birth. However, after 9 months there was no notable difference in the proportions of these cell populations. It has been shown before that primigravidae are more vulnerable to malaria [81], and from the present study results it could be assumed that it is more advantageous to have Pf+ IgG MBCs compared to Pf+ naïve B-cells, something that is also in line with the general assumption of how protective memory against a disease is formed. It would have been interesting to investigate presence of malaria parasites in placental tissue of all the mothers and examine if any correlations occur in factors such as pregnancy parity and parasite density. However due to limited resources and the scope of the study, these investigations could not be carried out.

## Conclusions

During the first 9 months of life in a malaria endemic area, babies not only develop Pf+ IgG MBCs and plasma cells/blasts but they also attain expansion of Pf+ atypical MBCs and non-IgG+ MBCs. The latter two sub-populations of cells occur at relatively high proportions in the adult mothers, indicating that traditional MBCs are not the only important memory B cells in the process of developing immunity against malaria.

## Additional file

**Additional file 1.** Additional figures.
