# Supplementary material for: Development of Plasmodium falciparum specific naïve, atypical, memory and plasma B cells during infancy and in adults in an endemic area
Source: Malar J. 2017 Jan 21;16:37. doi: 10.1186/s12936-017-1697-z (PMC5251336; doi:10.1186/s12936-017-1697-z)
Supplement: Supplementary file 1 — Additional file 1. Additional figures. [file 12936_2017_1697_MOESM1_ESM.doc]

**Additional information Lugaajju et al**

| **A**  **CD19**  **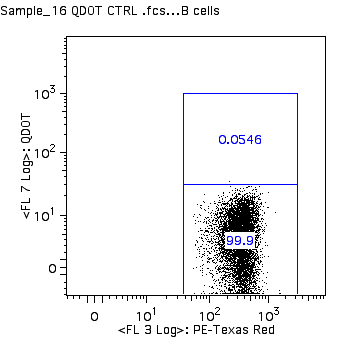**  **Q dot**  **Q dot alone** | **B**  **CD19**  **Q dot**  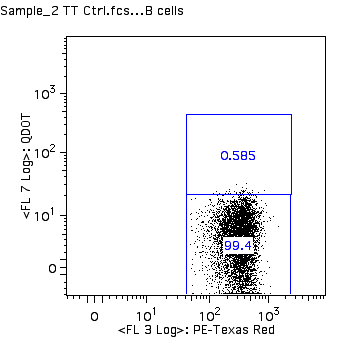  **Q dot GRBC** |
| --- | --- |
| **C**  **Q dot GiRBC**  **CD19**    **Q dot** | D  **CD19**  **Q dot GiRBC**  **Q dot** |
| Fig.1: Representative flow cytometry dot plot for Q dots alone (A), ghost uninfected RBC-Q dots (B), ghost infected RBC-Q dots (C) using a negative control sample and (D) ghost infected RBC-Q dots with a positive control sample. | |

| | | **FSC-H**  **Singlets**  **FSC-A**    **Lymphocytes**  **SSC**  **FSC-A** |  | | --- | --- | | **SSC**  **CD19**  **B-cells** | **CD20**  **Pf+ Plasma cells/blasts**  **CD27** | | **CD27**  **C**  **D**  **B**  **A**  **IgG** | A: Pf+ Non-IgG+ MBCs  B: Pf+ IgGMBCs  C: Pf+ Naïve B-Cells  D: Atypical MBCs |   Fig.2: Representative flow cytometry dot plot and gating procedure used to classify Pf+ B-cell sub-sets | | --- | --- | --- | --- | --- | --- | --- | |
| --- | --- | --- | --- | --- | --- | --- | --- |

| **A**  **CD19**  **Q dot** | **B**  **CD19**  **Q dot** |
| --- | --- |
| Fig.3: Example of flow cytometry dot plot showing Pf+ and Pf- specific B-cells for mother(A)-baby(B) pair at 9 months. | |
